# Immunization coverage of 12–23 months old children and its associated factors in Minjar-Shenkora district, Ethiopia: a community-based study

**DOI:** 10.1186/s12887-019-1575-7

**Published:** 2019-06-14

**Authors:** Alemayehu Gonie Mekonnen, Alebachew Demelash Bayleyegn, Esubalew Tesfahun Ayele

**Affiliations:** 1 Department of Nursing, College of Health Sciences, Debre Berhan University, Po. Box. 445, Debre Berhan, Ethiopia; 2 Department of Public Health, College of Health Sciences, Debre Berhan University, Po. Box. 445, Debre Berhan, Ethiopia

**Keywords:** Immunization coverage, Children aged 12–23 months, Minjar-shenkora district

## Abstract

**Background:**

Childhood vaccinations have been shown to be effective in protecting children against vaccine-preventable diseases. The systematic investigation of the causes of incomplete immunization is critical for the full immunization and develop health system interventions to improve immunization coverage. To date, no community-based immunization coverage assessment study was conducted in Minjar-shenkora district. Therefore, the aim of this study was to assess the immunization coverage and its factors among 12–23 months old children in Minjar-shenkora district, Ethiopia.

**Methods:**

Community-based cross-sectional study was conducted from September to November 2017. A total of 566 children aged 12–23 months and their mothers/caregivers were successfully interviewed using structured and pre-tested questionnaire. A stratified sampling technique was employed. Study participants were selected systematically. Data were entered into Epi data version 3.1 and exported into SPSS version 21 for analysis. Logistic regression analyses were done. A significant association was declared at a *p*-value less than 0.05.

**Results:**

Three fourth (75.6%) of 12–23 months old children were fully vaccinated. Incorrect appointment date (46.4%), the experience of child sickness with previous vaccination (35.2%) and disrespectful behavior of health professionals (14.3%) were the most common reasons cited by mothers/caregivers for incomplete vaccination of children. Being unmarried (AOR = 3.52, CI = 2.61, 9.15), not being a member of health development army (AOR = 3.31, CI = 2.01, 11.65) and traveling time greater than two hours on foot (AOR = 2.46, CI = 5.01, 17.18) were predictors of incomplete immunization.

**Conclusions:**

Child immunization coverage was still below the governmental plan of 90% in 2020. Being unmarried, not being a member of health development army and traveling time greater than two hours on foot were predictors of incomplete immunization. Strengthen health development army programmatic interventions in the community will improve child vaccination completion in the district. The issue of long travel time should be addressed by increasing the number of new vaccination sites/clusters in the district.

**Electronic supplementary material:**

The online version of this article (10.1186/s12887-019-1575-7) contains supplementary material, which is available to authorized users.

## Background

Childhood immunization against vaccine-preventable diseases has been the most cost-effective intervention among different public health interventions in developing countries. It is one of the most powerful preventive strategies to reduce deaths among children under five years old [1]. Globally, an estimated 1.5 million children die annually from diseases that can be prevented by immunization [2–4]. In Ethiopia, infectious and communicable diseases account for about 60–80% of the health problems [5] and about 16% under-five mortality has been attributed due to vaccine-preventable diseases [6].

In 1974, the World Health Organization (WHO) launched its expanded program on immunization (EPI) with the aim of controlling six childhood diseases: tuberculosis, diphtheria, pertussis, tetanus, polio and measles [7]. Accordingly, Ethiopia initiated the EPI program in 1980, and the national EPI aims to immunize all children between the ages of 0 and 23 months against eight vaccine-preventable diseases: tuberculosis, diphtheria, pertussis, tetanus, hepatitis B, and homophiles influenza, polio, and measles [8, 9]. Currently, the EPI program is expanding its service both in area and number of vaccines from time to time in Ethiopia [5]. The country also strictly follows the WHO recommendations for developing countries immunization schedule for child vaccination [10]. At present, there are ten EPI vaccines available in Ethiopia: BCG (Bacillus Calmette Guerin), measles, pentavalent, rotavirus, pneumococcus vaccine (PCV), and OPV (oral polio vaccine). Moreover, it is also directed in the implementation guideline to introduce inactivated poliovirus, measles-rubella, meningitis, and yellow fever vaccines for less than one-year-old children [11].

The government of Ethiopia and the regions have shown a strong commitment to EPI as evidenced by the expanded program of immunization services and commodities are provided free of charge in the health facilities and the service has been provided to the rural child population through health extension workers. Despite these efforts, the routine child immunization coverage is still not reached the target figures and realized the planned objectives [3]. According to Ethiopian demographic health survey report, the percentage of 12–23 months old children who received all basic vaccinations was 39% in 2016 [12]. As a result, in many parts of the country, the immunization coverage is less than the desired level of herd immunity to prevent the spread of eight EPI-targeted diseases. Similarly, there is a wide variation among regions regarding full immunization coverage ranging from 75.8% in Addis Ababa to 12.6% in Afar region [12]. Unfortunately, this variation of child vaccination status might be related to social, economic, geographic and cultural factors or the attitudes and capabilities of parents [13].

Numerous studies documented that home delivery, place of residence, mother’s knowledge about immunization, health workers household visit, distance to health institutions, poor perception about the benefit of immunization, and misconception about vaccine contraindication were predictors for child immunization [14–16]. Researchers have also demonstrated that the ways health workers perform their activities, the manner in which immunization activities are organized and services are delivered, the interaction between parents and health workers greatly influence the immunization coverage [17, 18]. Still, system-wide barriers are linked to incomplete vaccination or non-vaccination of children [11].

In order to improve full immunization of children against vaccine-preventable diseases, the underlying causes and mothers’/caregivers’ reasons not to immunize their children should be known, particularly in countries with the large numbers of unvaccinated children. The systematic investigation of the causes for the lack or drop out of immunization might help develop health system interventions to improve immunization coverage which in turn reduce vaccine-preventable diseases. To date, no community-based immunization coverage assessment study was conducted in Minjar-shenkora district. Therefore, the aim of this study was to assess the immunization coverage and its factors among 12–23 months old children in Minjar-shenkora district, Ethiopia.

## Methods and materials

### Study area and period

A community-based cross-sectional study was conducted from September to November 2017 in Minjar-shenkora district, Ethiopia. The district is located at 106-k meter far from Addis Ababa (the capital city of Ethiopia). According to the 2007 census, the district has an estimated total population of 155,436 in 2017. The district is sub-divided into 29 *kebeles (*sub-districts/cluster of villages). In the district, there are five EPI-clusters which routinely provide vaccination services. The total number of households was 38,859. About 5 % of the total population was children at the age of 12–23 months (*unpublished Minjar-shenkora health office report, 2017).*

### Study population

The study population consisted of all 12–23 months old children and their mothers/caregivers who lived within eligible households of selected kebeles in the study district. Mothers’/caretakers who had at least one living child of 12–23 months old were included in the study. However, those who were unable to respond or very sick were excluded.

### Sample size and sampling techniques

The sample size was determined by using single population proportion formula with the following assumptions: the proportion of fully vaccinated children in Amhara region to be 45.8% taken from Ethiopian demographic health survey 2016 report [12] with 95% confidence interval (CI), and margin of error to be 5%. Considering the 1.5 design effect (since the district was stratified into two strata), the total sample size of 573 households were selected.

A stratified sampling technique was employed. The whole study district was first stratified into urban and rural kebeles*.* From each stratum, six rural and one urban kebeles were selected by lottery method. In the second sampling unit (at the *kebele* level), family folders (list of households which was revised annually by health extension workers) were used as a sampling frame. These folders contained personal records of the household members: number of under-5 children, mothers’ date of delivery, the name of kebele *and ‘gote’*, development group leader, and their house number. The calculated sample size was proportionally allocated to (one urban and six rural) *kebeles* and the required numbers of households were selected using systematic random sampling technique. For those households having more than one eligible children, the youngest child was selected and in case of the twins both, children were included. If data collectors could not find any eligible mothers/ caregivers, they shifted to the next immediate household. Data collectors used the name of *kebeles,* their house numbers, and health extension workers for guidance.

### Data collection

A structured questionnaire was prepared in English then translated into Amharic (native language) and back into English to ensure consistency. The questionnaire was pre-tested using 5% of the sample size, and some modifications were made on the basis of the pre-test. The data were collected through face-to-face interview with mothers/caregivers and through a review of the vaccination cards. Mothers or caregivers were asked to show immunization cards for tracing the child’s immunization history. For those whose immunization cards were not available or lost, the mothers/caregivers were asked on the immunization status of their children. Sixteen diploma nurses participated in the data collection. Four supervisors supervised the data collection process in each day. Data collectors and supervisors were trained for two days before the actual data collection. Data completeness and consistency were checked by the investigators and supervisors.

### Measurements

The questioners were taken from Ethiopian demographic health survey 2016 and from peer-reviewed literature on immunization coverage [6, 16, 19–21]. The questioners comprise of four sections; socio-demographic characteristics, tools of vaccination coverage, and questions which explore mother’s/caregiver’s reasons for none or incomplete vaccination of children (Additional file 1).

### Operational definitions

#### Fully vaccinated

A 12–23 months old child who received the following vaccines; one dose of BCG, one dose of measles, two doses of Rota, at least three doses of Pentavalent, three doses of OP), and three doses of PCV.

#### Partially vaccinated

a 12–23 months old child who receive one dose of the above six vaccines.

#### Not vaccinated

a 12–23 months old child who didn’t receive any dose of the above six vaccines.

#### Dropout rate

the proportion of 12–23 months old child who failed to complete the vaccine.

The dropout rate of penta-3 was calculated as doses of Penta-1 administered minus doses of Penta-3 administered divided by doses of Penta-1 administered. The dropout rate of measles out of Penta-1 was calculated as doses of Penta-1 administered minus dose of measles administered divided by doses of penta-1 administered. The dropout rate of measles out of BCG was calculated as doses of BCG administered minus dose of measles administered divided by doses of BCG administered.

#### Vaccination coverage

the proportion of 12–23 months old children who took vaccination based on mother’s/caregivers’ report, and it was calculated as the number of fully immunized children divided by the number of surviving children (total sample size).

### Data processing and analysis

Data were checked for completeness and inconsistencies. Epi-data version 3.1 was used for data entry and data were exported to SPSS version 21. Descriptive statistics were computed. Logistic regression analysis was used to identify the relationship between dependent and independent variables. Those independent variables which were significant in bivariate analysis (*p*-value < 0.05) were entered into the multivariable analysis. In the final model, a significant association was declared at a p-value less than 0.05. And finally, the results were presented in texts and tables with adjusted odds ratio (AOR) and the corresponding 95% confidence interval.

### Ethical considerations

Ethical approval was obtained from the research and an ethical review committee of Debre Berhan University. Written informed consent was obtained from each study participant. All the information obtained from the study participants were kept confidential throughout the process of study, and the name of the participant was replaced by code. Withdrawal from the study at any point if they wished was assured.

## Results

### Socio-demographic characteristics

A total of 566 participants were successfully interviewed giving a response rate of 98.8%. About 35 % (35.9%) of the mothers/caregivers were in the age group of 25–29 years. The mean age of study participants was 28.26 (±3.82 SD). The largest proportions (94.2%) of mothers/caregivers were married and 67.7% of the respondents were living in rural areas. Regarding the educational level of the respondents, 41.4% of mothers/caregivers had no formal education while 43.6% had completed primary education. Sixty-six percent of respondents were members of the health development army (HDA) in the district. Twenty-seven percent of mothers/caregivers traveled less than 15 min to arrive at the vaccination site (Table 1).

**Table 1:** Sociodemographic status of respondents in Minjar-shenkora district, Ethiopia January 2017

Variables		Frequency	Percent
Age of mother’s/caregivers’	18–19	29	5.1
	20–24	149	26.3
	25–29	203	35.9
	≥30	185	32.7
Mother’s/caregivers’ residence	Rural	383	67.7
	Urban	183	32.3
Mother’s marital status	Unmarried	17	3.0
	Married	533	94.2
	Divorced/widowed	16	2.8
Mother’s educational status	No education	234	41.4
	Primary education	247	43.6
	Secondary education	64	11.3
	College education	21	3.7
Mother’s role in the community	Leader of HDA	79	13.5
	Member of HDA	373	66.4
	Not a member of HDA	114	20.1
Mother’s/caregivers’ occupation	Housewife/farmer	399	70.5
	Merchant	94	16.6
	Employee	54	9.5
	Daily laborer	19	3.4
Sex of the child	Male	291	51.4
	Female	275	48.6
Average distance to arrive at vaccination site	≤15 min	153	27.0
	> 15 to < 30 min	138	24.4
	≥30 min to ≤1 h	108	19.1
	> 1 h to < 2 h	118	20.8
	≥2 h	49	8.7

### Vaccination coverage

In this study, 92.9, 92.8%, 89.2, 91.9, and 85% of 12–23 months old children received OPV-3, Pentavalent 3, rota-2, PCV-3, and measles respectively. Four hundred twenty-eight (75.6%) of 12–23 months old children were fully vaccinated whereas 5.9% of children were not vaccinated at all. The remaining 18.5% of 12–23 months old children were partially vaccinated (Table 2).

**Table 2:** The proportion of 12–23 months old children receiving vaccines in Minjar-shenkora district, Ethiopia, January 2017

Vaccine	Frequency	Percent
BCG	529	93.5
OPV 0	95	16.8
OPV 1	534	94.3
OPV 2	530	93.6
OPV 3	526	92.9
Pentavalent 1	529	93.5
Pentavalent 2	532	94
Pentavalent 3	525	92.8
PCV 1	529	93.5
PCV 2	524	92.6
PCV 3	520	91.9
Rota 1	527	93.1
Rota 2	505	89.2
Measles	481	85
Fully vaccinated	428	75.6
Partially vaccinated	105	18.5
Not vaccinated	33	5.9

### Vaccination dropout rate

**Fig. 1:**
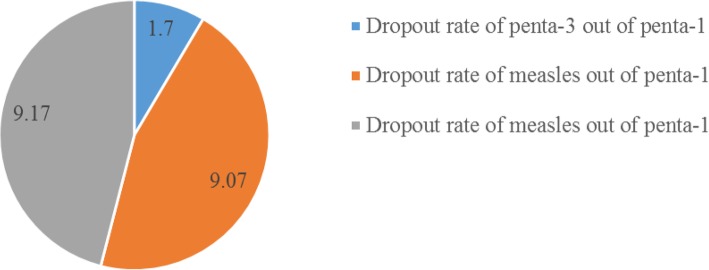
The vaccination dropout rate of 12–23 months old children in Mingar-shekora district, Ethiopia, January 2017

The figure shows the proportion of 12–23 months old child who failed to complete Penta-3 and measles vaccine. The calculated dropout rate for Penta-3 out of Penta-1, measles out of Penta-1 and out of BCG were 1.70, 9.07, and 9.07% respectively (Fig. 1).

### Mother’s/caregiver’s reason for incomplete vaccination of children

Incorrect appointment date (46.4%), stock out of vaccines (19.6%) were the most common reasons cited by mothers/caregivers for non/incomplete vaccination of their children. Among 105 mothers/caregivers who dropped out immunization, 35.2% of them declined for child immunization due to their experience of child sickness with previous vaccination. Missing appointment date (18.1%) and disrespectful behavior of health professionals (14.3%) were also reported as the major reasons for drop out for immunization (Table 3).

**Table 3:** Mother’s/caregiver’s response for incomplete vaccination of children in Minjar-shenkora district, Ethiopia, January 2017

Variables	Frequency	Percent
	*N*	%
Reasons for non/incomplete vaccination of children (*n* = 138)		
Incorrect appointment date	64	46.4
The absence/stock out of the vaccine in the vaccination site	27	19.6
The absence of a health professional in the health facility	16	11.6
Long waiting time	16	11.6
The vaccine will not be opened on the appointed date	12	8.7
My religion prohibits	9	6.5
Reasons for drop out for immunization (*n* = 105)		
The child was sick with previous vaccination	37	35.2
Missing the appointment date	19	18.1
Disrespectful behavior of health professionals	15	14.3
Ignorance of the use of vaccination	13	12.4
Long distance of the vaccination site	11	10.5
The health professionals serve inappropriately	10	9.5

### Multivariate analysis of factors associated with immunization coverage

Multivariate logistic regression showed that the three independent predictors of incomplete immunization. Unmarried women were 3.52 times more likely to incomplete child vaccination as compared to married women (AOR = 3.52, CI = 2.61, 9.15). Children were less likely to be vaccinated if the mothers were not a member of health development army. Mothers who were not a member of health development army were 3.31 times more likely to incomplete immunization as compared to mothers who were leaders of health development army (AOR = 3.31, CI = 2.01, 11.65). The 12–23 months old children living ≥2 h from vaccination site were significantly more likely to drop/fail in receiving vaccines as compared to children living < 15 min from vaccination site (AOR = 2.46, CI = 5.01, 17.18) (Table 4).

**Table 4:** The analysis of predictors of incomplete immunization of 12–23 months old children in Minjar-shenkora district, Ethiopia, January 2017

Variable	Categories	Vaccination status			
		Complete	Incomplete	COR(95% CI)	AOR(95% CI)
Mother’s occupation	Housewife/farmer	301	98	1.00	1.00
	Merchant	76	18	1.37(0.44, 5.43)	0.68(0.21,2.83)
	Employee	42	12	1.14(0.47, 2.98)	1.26(0.29,2.13)
	Daily laborer	9	10	0.29(0.19, 0.77)^*^	0.26(0.19,1.48)
Mother’s role in the community	Leader of HDA	68	11	1.00	1.00
	Member of HDA	293	80	1.69(0.54,6.72)	2.41(0.90,7.26)
	Not a member of HDA	67	47	4.34(1.53,9.31)	3.31(2.01,11.65)^*^
Mother’s marital status	Married	410	123	1.00	1.00
	Unmarried	8	9	3.75(1.72, 9.03)	3.52(2.61,9.15)^*^
	Divorced/widowed	10	6	2.00(0.14, 9.99)	2.27(0.23,7.98)
The average distance from home to the health facility	≤15 min	122	31	1.00	1.00
	> 15 - < 30 min	114	24	0.83(0.63, 2.72)	0.36(0.41,3.75)
	≥30 min - ≤1 h	81	27	1.31(0.44, 5.21)	1.62(0.52,8.13)
	> 1 h - 2 h	80	38	1.87(0.15, 0.81)	1.55(0.24,0.88)
	≥2 h	31	18	2.29(5.43, 12.02)	2.46(5.01,17.18)^*^

1 = reference, *p-value < 0.05

## Discussion

In this study, four hundred twenty-eight (75.6%) of 12–23 months old children were fully vaccinated. This figure was in line with studies reported in Ileje district, Tanzania (71.1%) [22], rural Mozambique (71.8%) [23], Lay-armachiho district (76.2%) [24] and Sinana district of southern Ethiopia (76.8%) [25]. It was also greater than the study conducted in Jigjiga district (36.6%) [26], Mecha district (49.3%) [16] and Mizan-aman town, southwest Ethiopia (42.2%) [27]. This immunization coverage variation might be due to the difference in access to vaccination services and community awareness towards child immunization. The quality of vaccination services might determine the likelihood of immunization service utilization and hence higher chances of vaccination completion. The dropout rate for Penta-3 out of Penta-1 was 1.7%. This finding was lower than the national strategic plan of 2020 which planned to reduce the dropout rate of Penta-3 by 2% [5]. This finding indirectly implies that the health extension program and HDA have become effective in increasing awareness about child immunization and in reducing the dropout rate by tracing immunization defaulters in the communities.

In the multivariate analysis, predictors of incomplete immunization were identified. Adjusting for other factors, unmarried women were 3.52 times more likely to incomplete child vaccination as compared to married women. Nevertheless, the previous studies did not report the statistical association between immunization status and marital status of women [21, 27]. The possible explanation could be the difference in cultural beliefs towards unmarried women in the study populations or married couples might discuss the vaccination of their child or husband might be involved in child care.

In this study, women’s role in the community was found to be significantly associated with immunization status of children. Mothers/caregivers who were not a member of health development army were 3.31 times more likely to incomplete immunization as compared to mothers who were leaders of HDA. This finding showed that health development army could be an important structure of community networking to scale up vaccination coverage. It also implies that discussing the benefit of vaccination during HDA meeting with health extension workers is a proven means to decide complete immunization of their child.

In this study, traveling time from home to the vaccination site was another predictive factor for incomplete vaccination. Children living ≥2 h away from the vaccination site were significantly more likely to drop/fail in receiving vaccines as compared to children living < 15 min from the vaccination site. This finding was in line with the study done in Khartoum, Sudan in which long distance to vaccination site was a predictor of incomplete child immunization [20]. In Laelay Adiabo district of Tigray region, mothers/caregivers who traveled more than 30 min to arrive at the vaccination site was associated with incomplete child immunization [19]. Okwaraji et al. also reported that a significant association between a traveling time to receive the vaccine and incomplete vaccination [21]. Similarly, the reasons for incomplete vaccination were associated with accessibility to the vaccination sites in Mozambique [23], Zimbabwe [9] and Debre-Markos town [11]. Thus, this further strengthened the argument that the time spent to reach the vaccination site expenses a high opportunity cost to mothers/caregivers by creating the need for multiple visits, especially when vaccine vials were not opened for a small number of children [19]. If mothers/caregivers traveled a long distance and failed to get the vaccination service, they might be enforced to default their children from completion of immunization.

## Conclusions

In this study, child immunization coverage was still below the governmental plan of 90% in 2020. This might be attributed to the incorrect appointment date, stock out of vaccines, the experience of child sickness with previous vaccination, and disrespectful behavior of health professionals. Being unmarried, not being a member of health development army and traveling time greater than two hours on foot were predictors of incomplete child immunization. Strengthen local community networking programmatic interventions (health development army) in the community will improve mothers’ awareness on the importance of immunization which again improves child vaccination completion in the district. The issue of long travel time should be addressed by increasing the number of new vaccination sites/clusters in the district.

## Additional files

Additional file 1: English version of consent form and questionnaire. (DOCX 20 kb)

## Data Availability

All data generated or analyzed during this study are included in this published article. In addition, part of the row datasets will be available from the corresponding author on reasonable request.

## Abbreviations

AOR: Adjusted Odds Ratio
BCG: Bacillus Calmette Guerin
CI: Confidence Interval
COR: Crude Odds Ratio
DOR: Dropout Rate
EPI: Expanded Program on Immunization
HBV: Hepatitis B Virus
HDA: Health Development Army
HEW: Health Extension Workers
PCV: Pneumococcal Conjugate Vaccine
