# Supplementary material for: Immunization coverage of 12–23 months old children and its associated factors in Minjar-Shenkora district, Ethiopia: a community-based study
Source: BMC Pediatr. 2019 Jun 14;19:198. doi: 10.1186/s12887-019-1575-7 (PMC6567439; doi:10.1186/s12887-019-1575-7)
Supplement: Supplementary file 1 — English version of consent form and questionnaire. (DOCX 20 kb) [file 12887_2019_1575_MOESM1_ESM.docx]

# **Annexes**

**1.1. Consent Form and Questionnaire**

Dear Madam -----------------------------

I am ------------ and currently working at Debre Berhan university. The aim of this study is assessing immunization coverage of 12–23 months old children and its associated factors in Minjar-Shenkora district, Ethiopia: a community-based study. The systematic investigation of the causes for the lack or drop out of immunization might help develop health system interventions to improve immunization coverage which in turn reduce vaccine-preventable diseases. This study will also enable to advise policy makers to improve full immunization of children against vaccine-preventable diseases.

Thus this interview is prepared for this purpose to get appropriate information on the topic. The information that will be obtained using this interview will be used only for research purpose. Confidentiality and anonymity is fully assured, as your name is not required and only the research team will have access to the results. It will not affect you in anyway, should you not take part in this study?

If yes continue to sign consent form If No, stop here

I have been informed that the purpose of this study is assessing assessing immunization coverage of 12–23 months old children and its associated factors in Minjar-Shenkora district, Ethiopia. I have understood that participation in this study is entirely voluntarily. I have been told that my answer to the question will not be given to anyone else and no reports of this study identify me in any way. I understood that participation in this study does not involve risks. I understood that Alebachew Demelash is a contact person if I have question about the study or about my right as a study participant.

Respondent’s Signature ___________Date___________Start interview.

Supervisor’s name ________________ signature _________

**Address of PI:** **Tell**: 0912603373 **e-mail:** [alebachew3373@gmail.com](mailto:alebachew3373@gmail.com)

**Instruction to the interviewer:** circle the number in front of the option based on the response

| **Sr.** | **Part I: Demographic Characteristics** | **Options** | **Remark** |
| --- | --- | --- | --- |
| 1.1 | Age of respondents (in years) | ------- |  |
| 1.2 | Marital status | 2= Married 1=Single/separated |  |
| 1.3 | Religion | 1= Muslim 2= orthodox 3= protestant |  |
| 1.4 | Educational status of woman | 1= Illiterate 2=primary education 3=secondary education 4= college education |  |
| 1.5 | Educational status of husband | 1= Illiterate 2= primary education 3=Secondary education 4= college education |  |
| 1.6 | Occupational status of women | 1= Housewife 2= Farmer 3= Merchant  4=Employee (government/private employee) |  |
| 1.7 | Occupational status of husband | 1= Farmer 2= Merchant  3=Employee (government/private employee) |  |
| 1.8 | Mother’s role in the community | 1=Leader of HAD 2=Member of HDA  3=Not a member of HDA |  |
| 1.9 | Mother’s/caregivers’ residence | 1=Urban 2= Rural |  |
| 1.10 | Sex of the child | 1=Male 2=Female |  |
| 1.11 | Average distance to arrive at vaccination site | 1=≤15 minutes 2=>15 to <30 minutes  3=≥30 minutes to ≤1 hour  4=>1 hour to <2 hours 5= ≥2 hours |  |
| **2.** | **Part II: Vaccination status measurements** | |  |
| 2.1 | Did your child vaccinated/ receive vaccines? | 1=yes 2= no If no skip to Q 3.1  3= start but not complete If start skip to Q 3.2 |  |
| 2.3 | Did your child receive BCG? | 1=yes 2= no |  |
| 2.4 | Did your child receive OPV vaccines? | OPV 0 1=yes 2= no  OPV 1 1=yes 2= no  OPV 2 1=yes 2= no  OPV 3 1=yes 2= no |  |
| 2.5 | Did your child receive pentavalent? | Pentavalent 1 1=yes 2= no  Pentavalent 2 1=yes 2= no  Pentavalent 3 1=yes 2= no |  |
| 2.6 | Did your child receive PCV? | PCV 1 1=yes 2= no  PCV 2 1=yes 2= no  PCV 3 1=yes 2= no |  |
| 2.7 | Did your child receive Rota? | Rota 1 1=yes 2= no  Rota 2 1=yes 2= no |  |
| 2.8 | Did your child receive Measles | 1=yes 2= no |  |
| **3. Part III: Mother’s/caregiver’s reason for incomplete vaccination of children** | | | |
| 3.1 | What is the reason for non-vaccination of children? (circle all possible answers) | 1=Incorrect appointment date  2=The absence/stock out of vaccine in the vaccination site  3=The absence of a health professional in the health facility  4=Long waiting time  5=The vaccine will not be opened on the appointed date  6=My religion prohibits  7=other specify ------------------ | |
| 3.2 | What is the reasons for drop/incomplete for immunization? (circle all possible answers) | 1=The child was sick with previous vaccination  2=Missing the appointment date  3=Disrespectful behavior of health professionals  4=Ignorance of the use of vaccination  5=Long distance of the vaccination site  6=The health professionals serve inappropriately  7=other specify ------------------ | |
